# Experimental Study on the Properties of Mortar and Concrete Made with Tunnel Slag Machine-Made Sand

**DOI:** 10.3390/ma15144817

**Published:** 2022-07-10

**Authors:** Yu Tang, Weichao Qiu, Dunwen Liu, Wanmao Zhang, Ruiping Zhang

**Affiliations:** 1 School of Resources and Safety Engineering, Central South University, Changsha 410083, China; tangyu12@csu.edu.cn (Y.T.); 205512136@csu.edu.cn (W.Z.); 2 Road & Bridge North China Engineering Co., Ltd., Beijing 101100, China; 215512141@csu.edu.cn (W.Q.); 215512132@csu.edu.cn (R.Z.)

**Keywords:** machine-made sand, mortar, stone powder, tunnel slag, gray-correlation analysis

## Abstract

Machine-made sand is gradually replacing natural sand to achieve sustainable development. Experimental studies and gray-correlation analysis were used to study the properties of tunnel slag machine-made mortar and concrete. The properties of machine-made mortar with different stone powder content were analyzed through experiments. By analyzing the performance of machine-made sand concrete with equal amounts of cement replaced by stone powder, the optimum replacement ratio is obtained. Gray-correlation analysis was used to compare the degree of influence of fineness modulus and stone powder content on the performance of concrete. Scanning electron microscopy (SEM) and X-ray diffractometry (XRD) were used to analyze the microstructure of tunnel slag sand concrete. The test results showed that the flexural and compressive strengths of the machine-made sand concrete were greater than the standard sand with the same stone powder content. The 28-day flexural and compressive strengths had a maximum difference of more than 30%. The best stone powder content of the machine-made mortar is in the range of 5% to 8%. When the replacement cement content of stone powder is about 6%, the mechanical and working properties of machine-made sand concrete achieve the optimal state. The lower the stone powder content, the closer the mechanical and working properties of machine-made sand concrete and river sand concrete. The correlation between the performance of machine-made sand concrete and fineness modulus is the largest. When the stone powder content is low, it has almost no effect on the compressive strength of concrete. The results point out the direction for the quality control of tunnel slag machine-made sand concrete.

## 1. Introduction

Concrete is the most widely used building structure material today [1]. With the acceleration of urbanization and the rapid development of highways and railroads, the demand for concrete materials is gradually increasing [2]. In recent years, natural sand has been over-exploited, and environmental protection is gradually strengthened. The lack of sand resources in China has led to the rapid development of the building materials industry, which has begun to use a large amount of machine-made sand [3,4,5]. Studies have shown that compared with natural sand, artificial sand has the advantages of abundant resources and controllable quality [6]. Machine-made sand is a kind of artificial sand made of stone crushed and screened by a sand-making machine, and the particle size is mostly less than 4.75 mm. The raw material of machine-made sand mainly includes tunnel excavation waste rock [7], etc. Tunnel waste rock is usually piled up in nearby dumps for landfill, which is not conducive to sustainable development. The crushed stone and stone powder in tunnel waste rock can be recycled to produce artificial sand [8].

The most obvious difference between the machine-made sand and natural sand is that the machine-made sand will produce stone powder with particle size less than 0.075 mm. The limit value of stone powder is 10% according to the national standard in China, but the content of artificial sand in actual production is usually as high as 10–20% [6]. The stone powder in the artificial sand is different from the mud powder in natural river sand. It is the same as the lithology of the parent rock and the mineral composition in the production raw materials, and its particle size distribution and differs greatly from the mud powder. In addition, the stone powder can constitute the microfine gradation of machine-made sand, which is an important part of the sand. It has an important influence on the strength, workability and durability of concrete [9,10,11]. This has limited the popularization of machine-made sand concrete to a certain extent. At present, there are more studies at home and abroad on the influence of stone powder content on concrete properties [12,13,14]. There have been conflicting statements about the role of stone powder content in the performance of concrete. Some scholars believe that the appropriate stone powder content can improve the workability and strength of machine-made sand concrete [15,16,17,18]. Others believe that the increase of stone powder content will lead to the decrease of workability and strength [19]. Sua Iam et al. [20] found that the compressive strength of concrete was best when the stone powder content was 10%. Diab et al. [21] investigated the compressive strength of concrete with stone powder content of 0%, 5%, and 10% to 25%. The strength decreased with increasing stone powder content. However, the decrease in compressive strength was not significant until the stone powder content was below 10%. Shen et al. [22] proposed that when the stone powder content was 7.5%, the highest compressive strength of concrete was achieved. Most scholars found that the cube compressive strength test of machine-made sand concrete was better than that of concrete with natural sand. This is because the machine-made sand is rougher and has more angles than the natural sand, which can increase the friction and better bond with the cement. This increases the bond between the two and improves the strength of the concrete. However, the strength is affected by many factors, which may lead to different results. The effect of stone powder on the concrete properties needs further study, especially in tunnel slag machine-made sand concrete.

Unlike natural sand, the mineral and chemical composition of stone powder is the same as that of the parent rock of machine-made sand. The effect of stone powder content on mortar properties has been extensively studied [23,24]. Chong et al. [25] proposed that stone powder can shorten the setting of cementitious materials. Pliya et al. [26] suggested that 5% stone powder content increased the compressive strength of mortar, but the flexural strength of mortar decreased when the content exceeded 10%. Mohammadi et al. [27] suggested that when the limestone powder content increased from 7.5% to 12%, the physical properties of mortar remained similar. Khaleel et al. [28] showed that the rheological and mechanical properties of fresh mortar were improved when the stone powder content was 10%. Dave et al. [29] found that the best mechanical properties and resistance to chloride ion penetration of mortar samples were obtained when limestone powder content was 7.5–10%. Temiz et al. [30] found that the compressive strength of mortar decreased with increasing limestone powder content. Ji et al. [31] suggested that the early strength of mortar increased when limestone powder content was 20%. Rizwan et al. [32] proposed that the strength of self-compacting mortar was improved, and the pore size decreased when the limestone powder content was 20%. Due to the restriction on the development and use of natural sand, the machine-made sand has received more and more attention [33]. It is gradually replacing natural sand in the manufacture of concrete and other cement-based materials [34,35,36]. The scarcity of river sand resources in eastern China has restricted the development of local engineering construction. However, tunnel slag disposal resources are abundant, and tunnel slag machine-made mortar and concrete are relatively little used in engineering practice [8,37]. In addition, due to the fluctuation of stone powder content during the production of machine-made sand, there are fewer studies on the influence of sand quality changes on the properties of mortar and concrete. Therefore, the performance of tunnel slag machine-made mortar and concrete needs to be further investigated.

This study focuses on the following aspects of tunnel slag sand concrete. Experimental studies and gray-correlation analysis were used to study the properties of tunnel slag mortar and concrete. The flexural strengths of standard sand and machine-made sand concrete with different stone powder contents were analyzed through experimental studies. The workability and mechanical properties of the concrete with the same amount of cement replaced by stone powder were analyzed to find out the best stone powder replacement ratio. The correlation between fineness modulus and stone powder content on the slump and strength of the machine-made sand concrete was calculated. The degree and law of influence of different factors on the performance of machine-made sand concrete were analyzed. Scanning electron microscopy (SEM) and X-ray diffractometer (XRD) were used to analyze the microstructure of tunnel slag sand concrete.

## 2. Materials and Methods

### 2.1. Materials

In this study, the raw material cement was P.O 42.5 grade ordinary silicate cement of Changshan South brand. Two grades of 5–10 mm and 10–20 mm were used as coarse aggregates for the test. The chert gravel was mixed into 5–20 mm continuous graded gravel in the ratio of 4:6. The surface bulk density of mixed coarse aggregate was 1456 kg/m^3^, and the crushing value was 10.2%. The aggregates were washed manually to clean the powder on the surface. The fine aggregate for the test is the machine-made sand obtained by artificial crushing of the parent rock. The content of particles less than 75 μm in the machine-made sand was fixed at 2.4%. The fineness modulus is 2.9. The fly ash is grade II fly ash. The external additive is polycarboxylic acid high performance water reducing agent. 

**Figure 1:**
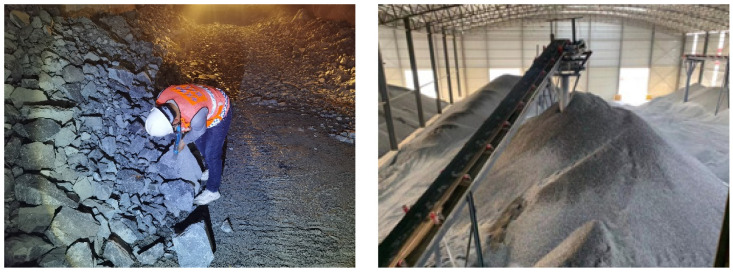
Tunnel slag and machine-made sand.

The source of the base material is the tunnel slag produced from the inspected and qualified tunnel working face. Tunnel slag and machine-made sand produced are shown in Figure 1. According to the geological conditions in the design and construction drawings, the source of the parent material was initially determined by combining the lithology, strength and weathering degree of the surrounding rock. When unqualified parent rock is encountered, the recycled slag in this section is directly discarded. The strength of the parent rock is quickly tested by rebound method to ensure the source of the parent material is reliable. According to the central laboratory exploration core samples for testing, the compressive strength test results are shown in Table 1. According to the Chinese railway industry standard “TB10424-2018” for crushed rock parent rock strength is greater than 1.5 times the design strength of the concrete compressive strength grade requirements. The test determined that the tunnel slag meets the strength requirements of C40 and below concrete for parent rock. Three types of parent materials were selected and sent to the testing center of the Geological Engineering Survey Institute for rock and mineral identification. The results of the appraisal all belong to sandstone. The composition of the parent material is mainly quartz, followed by rock chip, feldspar and black mica.

### 2.2. Experiment Design 

The biggest problem in the practical application of tunnel slag sand concrete is the poor working performance. Therefore, it is of utmost importance to study the compatibility of the machine-made sand concrete. Compared with natural river sand, the appearance morphology, angularity and fineness modulus of the machine-made sand have large differences. The effect of stone powder content in the machine-made sand on the workability of mortar and concrete is very significant. For now, the research on the machine-made sand concrete properties is still controversial. In addition, most studies are limited to limestone [37] and granite machine-made sand, and there is little research on siltstone concrete. The effect of stone powder content on siltstone mortar and concrete is still unknown.

The mechanical properties of mortar are related to whether the structure of mortar can meet the requirements of engineering use. The flexural and compressive strengths of the mortar of standard sand and machine-made sand with different stone powder contents were studied. In the preliminary preparation of the experiment, the standard sieve with minimum size of 0.075 mm was used to obtain the pulverized sand rock powder by sieving the machine-made sand step by step. Then the amount of cement was replaced by the equal mass of stone powder to prepare standard sand mortar. The test design of standard sand mortar is shown in Table 2. The machine-made sand mortar was prepared by replacing the sand with the equal mass of stone powder. The test design of machine-made sand mortar is shown in Table 3. All raw materials used for the test were the same batch to ensure the accuracy of the test results as much as possible.

Machine-made sand concrete mixes with equal amount of cement replaced by stone powder were used to study the effect of stone powder replacement of cement on the properties of concrete. After several mixing sessions, the slump, slump expansion and compressive strength of the machine-made sand concrete were recorded. The performance of the machine-made sand concrete with equal amount of cement replaced by stone powder was analyzed to arrive at the optimum stone powder replacement ratio. To investigate the degree of influence of fineness modulus and stone powder content on the properties of concrete, we prepared river sand concrete with different fineness modulus. Machine-made sand concrete with different fineness modulus and stone powder content was prepared. The fineness modulus of river sand and machine-made sand was taken from 2.4 to 3.0, adjusted by 0.1 for each level. In addition, the stone powder content was taken from 0 to 10%, adjusted by 2% for each level. The test performance is based on workability and mechanical properties. The workability test indexes are slump and slump expansion, and the mechanical property indexes are 3-day, 7-day, 28-day and 56-day compressive strengths. 

### 2.3. Analysis Methods 

The degree of influence of the index such as fineness modulus of tunnel slag machine-made sand on the concrete properties is different. The properties of concrete with different stone powder content and fineness modulus were tested. Gray-correlation analysis was used to calculate the correlation between the stone powder content, fineness modulus and the slump, strength of concrete [6]. 

Gray-correlation analysis is commonly used in concrete performance studies. In the analysis to investigate the effect of machine-made sandstone powder content, fineness modulus and concrete workability and mechanical properties. Two subfactors are tunnel cavity slag stone powder content and fineness modulus. Three parent factors are concrete slump, 7-day and 28-day compressive strengths. Each parent factor has two correlation degrees corresponding to the two subfactors. The calculation steps are as follows.

1. Determine the data column.

Determine the reference data column, denoted as $x_{0}$. The parent sequence $x_{0}$ can be expressed as $x_{0}$ = ($x_{0}\left(1\right),x_{0}\left(2\right),$…$,x_{0}\left(k\right)$), where $x_{0}\left(k\right)$ is the *k*th case.

2. Dimensionless processing.

The dimensionless processing can be used to divide each number in the series by the maximum value in the series in turn.

3. Calculate the correlation coefficient and correlation degree.

There are *k* comparison subsequences$\;x_{1}$,$\;x_{2}$, …,$x_{k}$, and the correlation coefficient is calculated as follows.

(1) $${\xi \;}_{i}=\frac{\underset{i}{\min}\underset{k}{\min}\left|x_{0}\left(k\right)-x_{i}\left(k\right)\right|+\;\rho \underset{i}{\max}\underset{k}{\max}|x_{0}\left(k\right)-x_{i}\left(k\right)|}{\left|x_{0}\left(k\right)-x_{i}\left(k\right)\right|+\;\rho \underset{i}{\max}\underset{k}{\max}|x_{0}\left(k\right)-x_{i}\left(k\right)|},$$

where ρ is the resolution, ρ=0.5.

The degree of correlation is calculated as follows.

(2) $$r_{i}=\frac{1}{N}\sum_{k=1}^{N}{\xi \;}_{i}\left(k\right),$$

4. Construct the matrix.

The correlations of *m* parent factors with the corresponding *n* subfactors are obtained. The correlation matrix *R* allows for the dominance analysis of the parent and child sequences.

(3) $$R=\left(\begin{array}{cc} \begin{array}{cc} r_{11} & r_{12}\\ r_{21} & r_{22} \end{array} & \begin{array}{cc} \cdots & r_{1n}\\ \cdots & r_{2n} \end{array}\\ \begin{array}{cc} \cdots & \cdots \\ r_{m1} & r_{m2} \end{array} & \begin{array}{cc} \ddots & \cdots \\ \cdots & r_{mn} \end{array} \end{array}\right),\;$$

### 2.4. Test Methods 

**Figure 2:**
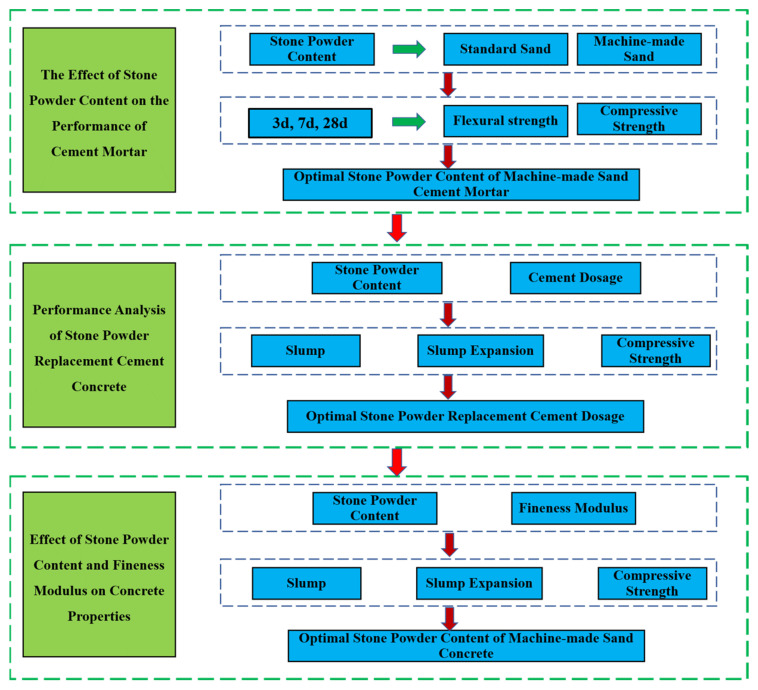
Flow chart of this study.

**Figure 3:**
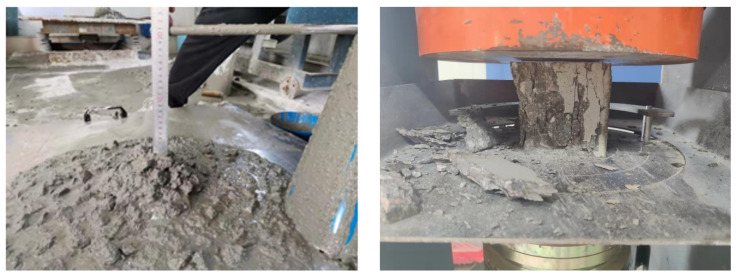
Test process diagram.

The strength grade of concrete is C35, and the concrete mix ratio design of concrete is shown in Table 4. It is required that the concrete has good workability and mechanical properties. The workability test indexes are slump and slump expansion, the operation is carried out in accordance with the Chinese standard GB/T50080-2016 “ordinary concrete mix performance test method standard” slump test. The test indexes of mechanical properties are compressive strengths, and the specimen size is 150 mm × 150 mm × 150 mm concrete test block. Additionally, the test method is conducted in accordance with the compressive strength test in Chinese standard GB/T50081-2002 “Standard of Mechanical Properties of General Concrete Test Methods”. The study process is shown in Figure 2. The field testing is shown in Figure 3.

## 3. Results and Discussion

### 3.1. Effect of Stone Powder Content on the Properties of Standard and Machine-Made Sand Mortar

**Figure 4:**
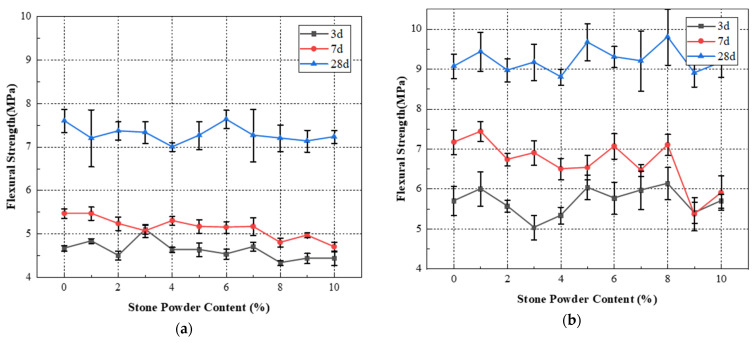
Flexural strength of mortar versus stone powder content curve: (**a**) standard sand; (**b**) machine-made sand.

**Figure 5:**
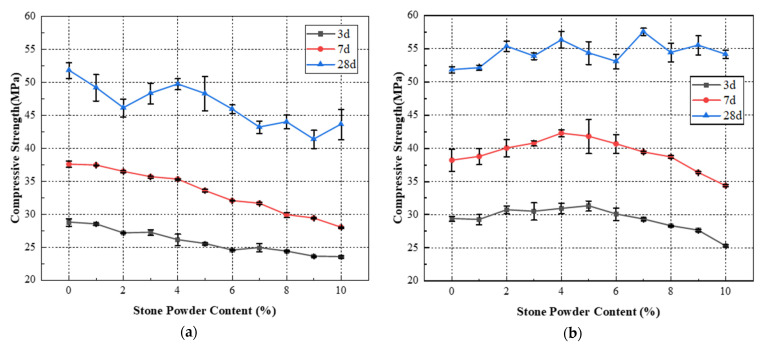
Compressive strength of mortar versus stone powder content curve: (**a**) standard sand; (**b**) machine-made sand.

The flexural and compressive strengths of standard sand and machine-made sand mortar with different stone powder contents were studied experimentally. The average value of the three test results was taken as the strength value, and its variation law was summarized. The test results of standard sand and machine-made sand mortar with different stone powder content are shown in Table 5, Table 6, Table 7 and Table 8. The flexural and compressive strengths of standard sand and machine-made sand mortar in relation to stone powder content are shown in Figure 4 and Figure 5.

From Table 5 and Table 6 and Figure 4, it can be seen that the flexural strengths of standard sand mortar at all ages tend to decrease with the increase of stone powder content. The flexural strength fluctuated in the range of 0% to 10%, but the variation was small. When the content is 6%, the 28-day flexural strength of mortar reaches the maximum. The maximum flexural strength of 3-day is reached when the content is 3%. The strength at the same age increased by 13.3% compared with that at 2%. The flexural strengths of the machine-made sand mortar at all ages fluctuate with the increase of the stone powder content, with a general trend of decreasing, then increasing and then decreasing. The flexural strength of the mortar reaches the maximum value when the content of stone powder varies from 5% to 8%. The flexural strength value of the machine-made sand is greater than that of the standard sand with the same stone powder content, and the maximum difference of 28-day flexural strength reaches 36.1%. This is because the stone powder has the function of filling pores and retaining water and thickening. The appropriate increase of stone powder content helps to reduce the porosity of colluvium, and can effectively reduce the risk of colluvium segregation and water secretion. Thus, it improves the structure of the internal cement–aggregate transition zone of the mortar and increases the flexural strength. With the continuous increase of stone powder content, the density and water retention of the mortar start to decrease. The specific surface area of the aggregate and the proportion of expansive clay minerals also increase. More cementitious material is needed to maintain the original adhesive strength between cement paste and aggregate. The structure of the cement–aggregate transition zone is weakened, and the flexural strength of the cement-sand is subsequently reduced.

In addition, it should be noted that the 7-day, 14-day and 28-day flexural strength maxima of the cement sands do not correspond to the same stone powder content. This is due to the fact that the stone powder particles provide a favorable surface for the hydrated silicate gel to nucleate and grow during the early hydration of the cement. It accelerates the hydration of silicate cement and plays a microcrystalline nucleation acceleration effect. However, this accelerating effect is only useful for the early strength formation of concrete. It does not help much for the later hydration of cement, so it is beneficial to increase the stone powder content appropriately for the early strength formation of the cement.

It can be seen from Table 7 and Table 8 and Figure 5 that the compressive strengths of standard sand mortar show a general decreasing trend with the increase of stone powder content. The 28-day compressive strength fluctuated in the range of 0% to 4%, but the variation was small. The 28-day compressive strength of mortar reached the maximum at 4% stone powder content. The 3-day and 7-day compressive strengths of the machine-made sand showed a trend of increasing and then decreasing with the increase of stone powder content. Twenty-eight-day compressive strength fluctuated a little with the increase of stone powder content, but the overall trend was increasing. The 3-day and 7-day compressive strengths of mortar reached the maximum value when the content was 4% to 5%. The 28-day compressive strength of mortar reached the maximum value at 7% stone powder content. This is in general agreement with the optimal stone powder content proposed by Pliya [26] and Dave et al. [29]. The compressive strength values of the machine-made sand mortar were greater than those of the standard sand. Additionally, the maximum difference in 28-day flexural strength reached 33.1%. This is because mortar is a typical porous material. The pores are mainly filled by free water and a part of unhydrated cement fine particles and a small amount of stone powder particles. Appropriate increase of stone powder content helps to improve the compactness of mortar, which makes the cement particles and pore free water originally filled in the pores participate in the hydration reaction. The compressive strength of the mortar increases accordingly. However, the excessive stone powder content destroys the reasonable particle gradation of the cement–aggregate. The reduction of coarse particles weakens the skeletal role of the aggregate, which leads to the reduction of the compactness and compressive strength of the mortar.

### 3.2. Analysis of the Performance of Concrete with Equivalent Amount of Cement Replaced by Stone Powder 

**Figure 6:**
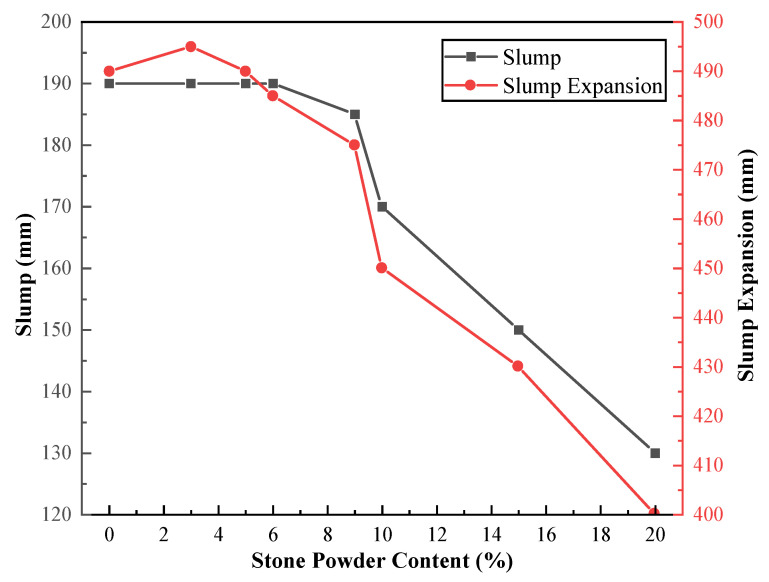
Concrete slump and slump expansion versus stone powder content curve.

**Figure 7:**
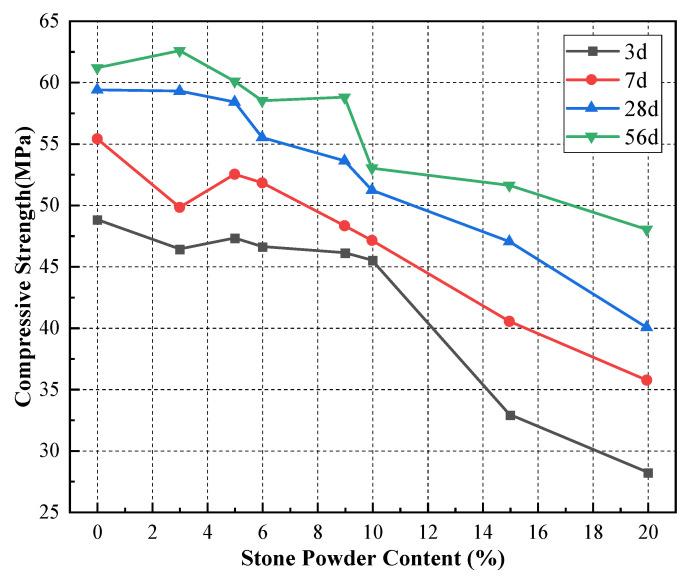
Concrete compressive strength versus stone powder content curve.

To investigate the effect of stone powder replacing equal amounts of cement on the properties of concrete, we tested the concrete mixes of machine-made sand with different amounts of stone powder blended to replace cement. The results of slump, slump expansion and compressive strength tests the concrete are shown in Table 9. The curves of concrete properties versus stone powder content are shown in Figure 6 and Figure 7.

As can be seen from Figure 6, the slump of the machine-made sand concrete tends to decrease as the proportion of stone powder replacing cement increases. When the stone powder content is within 9%, the slump and slump expansion of concrete do not change significantly, and the compatibility is best at this time. When the content exceeded 9%, the slump and slump expansion of concrete decreased significantly, with a maximum drop of 30%. This is due to the fact that the appropriate amount of stone powder increases the slurry content, which makes up for the shortcomings of the grain shape of the machine-made sand and improves the workability of the mix. However, as the specific surface area of stone powder is much larger than that of the machine-made sand. Excess stone powder increases the total specific surface area of solids and increases the water requirement of slurry. In addition, water use does not increase, which leads to a reduction in concrete slump. The pumping performance as well as the workability of the machine-made sand concrete is growing worse. Therefore, for tunnel slag siltstone machine-made sand, the negative effect of stone powder on concrete flowability is greater than the positive effect. The larger amount of stone powder admixture is detrimental to the workability of concrete. Its optimal stone powder replacement amount is around 6%, and should not exceed 9%.

As can be seen from Figure 7, the compressive strength of concrete at different ages gradually decreases when the percentage of stone powder replacing cement increases from 0% to 20%. The amount of change in compressive strength of stone powder content within 10% was less than 10% to 20%, and the 3-day compressive strength decreased by 6.8% and 38.0%, respectively. When the stone powder content was within 5%, the compressive strength of concrete at 28-day and 56-day changed less and maintained a high level. The compressive strength of concrete at 7-day decreases and then increases, reaching a peak at 5% of stone powder content. It indicates that the effect of stone powder content on the compressive strength of the concrete of machine-made sand at different ages is basically the same. The effect of stone powder on the compressive strength of machine-made sand concrete has both advantages and disadvantages. The benefit is that the filling effect of stone powder improves the interfacial transition zone between aggregate and cement paste. The crystalline nucleation promotes the early hydration reaction and effectively improves the concrete strength. In addition, the disadvantage is that with the increase of stone powder in excess, the closest accumulation of aggregates is destroyed, which makes the concrete strength decrease. As can be seen from Figure 7, the compressive strength of siltstone machine-made sand concrete at all ages is at a high level when the percentage of stone powder replacement is not greater than 9%. This is similar to the results of the study by Sua Iam [20] and Shen et al. [22] on the stone powder content in the machine-made sand and the compressive strength of concrete.

### 3.3. Effect of Fineness Modulus and Stone Powder Content on Concrete Properties 

**Figure 8:**
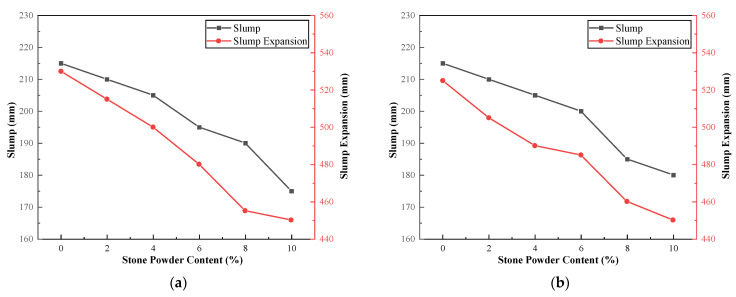

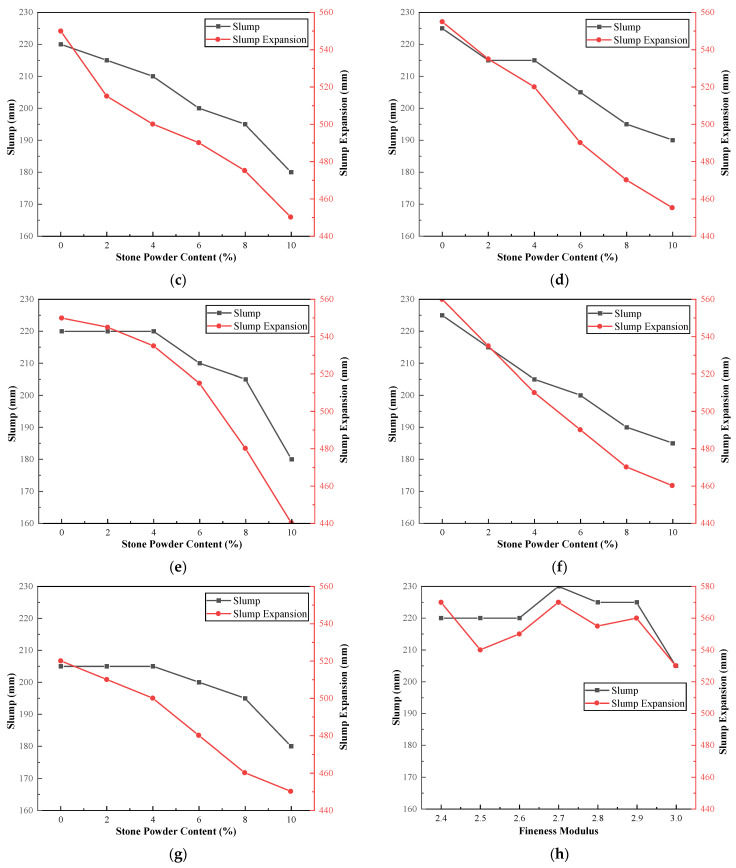
Concrete workability versus fineness modulus and stone powder content curve: (**a**) fineness modulus 2.4; (**b**) fineness modulus 2.5; (**c**) fineness modulus 2.6; (**d**) fineness modulus 2.7; (**e**) fineness modulus 2.8; (**f**) fineness modulus 2.9; (**g**) fineness modulus 3.0; (**h**) river sand concrete.

**Figure 9:**
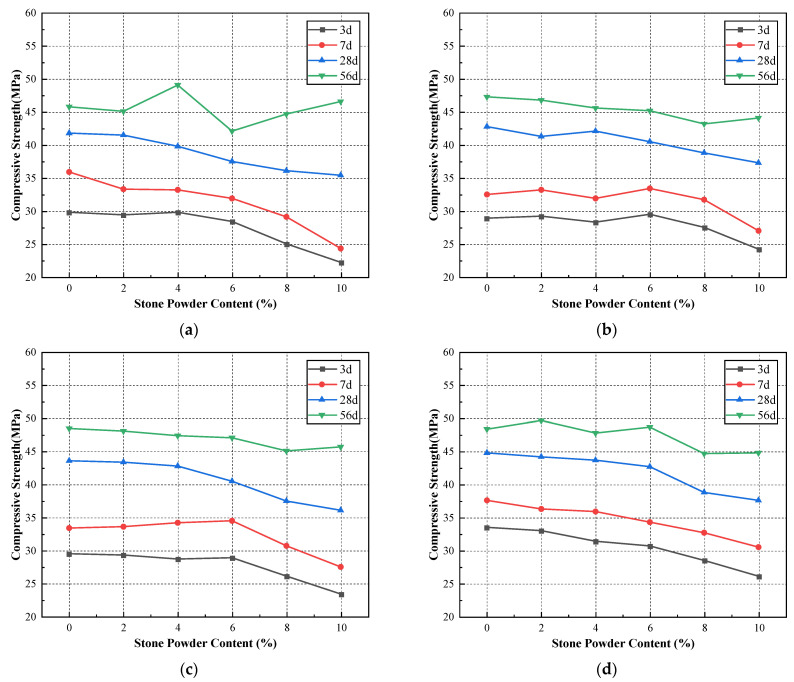

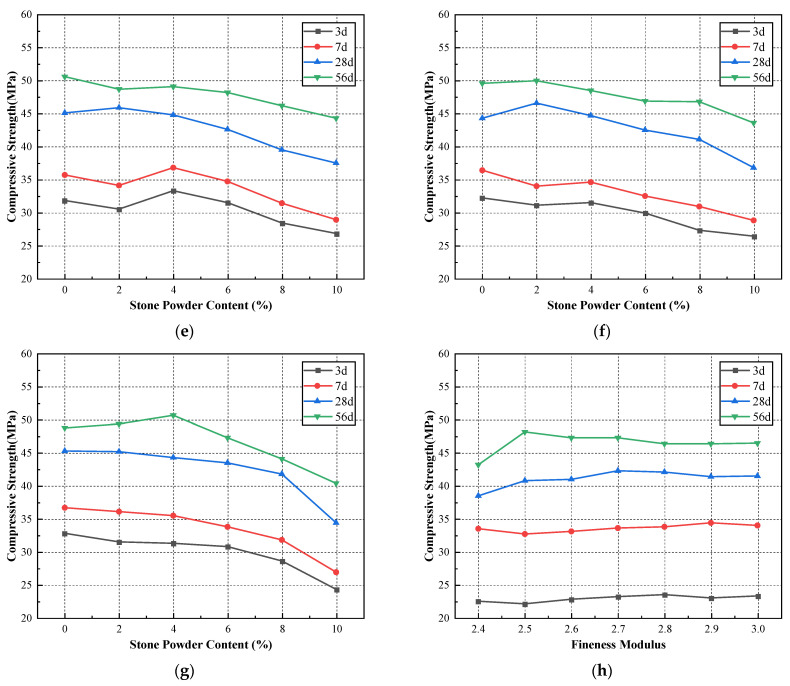
Mechanical properties versus fineness modulus and stone powder content curve: (**a**) fineness modulus 2.4; (**b**) fineness modulus 2.5; (**c**) fineness modulus 2.6; (**d**) fineness modulus 2.7; (**e**) fineness modulus 2.8; (**f**) fineness modulus 2.9; (**g**) fineness modulus 3.0; (**h**) river sand concrete.

To investigate the degree of influence of fineness modulus and stone powder content on the properties of concrete, we tested river sand and machine-made sand concrete mixes with different fineness modulus and stone powder content. The results of the tests for river sand concrete with different fineness modulus are shown in Table 10, and the results of the tests for machine-made sand concrete with different fineness modulus and stone powder content are shown in Table 11. The relationship curves between fineness modulus and stone powder content and concrete workability and mechanical properties are shown in Figure 8 and Figure 9.

The slump and slump expansion of the concrete have the same trend. The slump gradually decreases with the increase of stone powder content, which is consistent with the findings of the Section 3.1 and Section 3.2. This is mainly due to the rough and angular surface of the particles of the machine-made sand, which also requires more cement paste than the smooth surface and rounded particles of river sand. Additionally, this indirectly leads to insufficient content of paste to wrap the coarse aggregate, thus showing a decrease in slump and workability in general. The workability of the machine-sand concrete does not change significantly with increasing fineness modulus. The workability of concrete with a fineness modulus of about 2.7 is optimal. When the fineness modulus exceeds 2.9, the workability of concrete decreases significantly. The working performance of river sand concrete changes similarly with the fineness modulus. The coarser the sand, the greater the slump of concrete; the finer the sand, the smaller the slump of concrete. The slump of river sand concrete can reach up to 230 mm, which is greater than that of the concrete of the machine-made sand under the same conditions. The gradation of river sand used in this study is not much different from that of the machine-made sand. However, the natural sand angularity is significantly smaller than that of the machine-made sand, the fluidity will be better. Therefore, the slump of the river sand concrete will be slightly larger than that of the machine-made sand.

The compressive strengths of the machine-made sand concrete at different ages have a similar trend. The compressive strength of concrete tends to decrease roughly with the increase of stone powder content. The compressive strength of the machine-made sand concrete does not change significantly with the increase of fineness modulus. When the fineness modulus was around 2.7, the compressive strength of the concrete was the maximum, and the mechanical properties were the best at this time. The compressive strength of machine-made sand concrete decreases significantly with the increase of stone powder content when the fineness modulus is 3.0. With the increase of fineness modulus, the change in compressive strength of river sand concrete is very small. When the fineness modulus is 2.5, the maximum 56-day compressive strength of river sand concrete is 48.2 MPa, while the maximum 56-day compressive strength of machine-made sand concrete reaches 50.7 MPa. When the fineness modulus of machine-made sand concrete is greater than 2.7 and the stone powder content is less than 4%, the mechanical properties of machine-made sand concrete are better than those of river sand concrete. Compared with river sand, the machine-made sand particles are irregular in shape, angularity and rough surface, and the bite between the machine-made sand aggregates and the bond strength between cement and fine aggregates are greater. Thus, when the right amount of stone powder, the machine-made of sand concrete compressive strength is greater. The test results show that under the premise of controlling the amount of stone powder, the concrete prepared by tunnel slag machine-made sand has higher strength and better structure than river sand concrete.

### 3.4. Gray-Correlation Analysis of the Concrete Performance 

Based on the results of the tests of the machine-made sand concrete with different fineness modulus and stone powder content in Table 11. The values of sub-series factors and parent series factors of tunnel slag sand concrete are primed. According to the gray-correlation analysis steps of the calculation, the tunnel slag machine-made sand concrete performance correlation matrix *R* was obtained.

(4) $$R=\left(\begin{array}{cc} 0.5818 & 0.9246\\ 0.5883 & 0.9341\\ 0.5816 & 0.9380 \end{array}\right),$$

Comparing the correlation matrix *R* of the properties of the machine-made sand concrete, $r_{12}>r_{11}$ indicate that the correlation between slump and fineness modulus of machine-made sand concrete is the largest. The higher the fineness modulus of sand is, the poorer the cohesiveness and water retention of concrete are, and the phenomenon of segregation and water secretion is easy to occur. As the fineness modulus of sand decreases, the workability of concrete becomes better and better. However, after reducing to a certain value, the concrete becomes loose, the fluidity becomes smaller, and the workability becomes worse and worse. The addition of stone powder has positive and negative effects, on the one hand, the admixture of stone powder can be filled with the cementitious material with each other, filling the gap between the particles. It increases the content of free water in the cement paste, and promotes the concrete to enhance the fluidity. However, on the other hand, the specific surface area of stone powder is large, the increase of stone powder admixture makes the water demand also increased sharply, thus reducing the fluidity. $r_{22}>r_{21}$,$r_{32}>r_{31}$ indicate that the correlation between the compressive strength and fineness modulus of the concrete is the highest. Fineness modulus is the main influencing factor on the strength of machine-made sand concrete. The greater the fineness modulus of aggregate is, the greater the contact area between aggregate and cement slurry, the stronger the bond is. Concrete is less prone to damage between aggregates when it is pressed, showing higher compressive strength. $r_{21}>r_{31}$,$r_{32}>r_{22}$ indicate that comparing the compressive strength of concrete at different ages, the 7-day compressive strength of concrete has a greater correlation with the fineness modulus. The correlation between stone powder content and 28-day strength of concrete was greater. The hydration degree of fly ash in concrete is low in the early stage, and the enhancing effect of mineral admixtures on concrete strength has not been reflected. Therefore, the early strength of concrete is more affected by fineness modulus. $r_{i1}<0.6$ (*i* = 1,2,3) indicate that there is no obvious correlation between concrete performance and stone powder content. When the content of stone powder is less than about 10%, stone powder has little effect on the compressive strength and slump of concrete. As for the failure characteristics of concrete, the most frequent parts of failure are the interface between aggregate and cement slurry, and most of them are the failure mode of bond surface. Stone powder concrete has little influence on bonding effect. Therefore, the improvement effect of stone powder on the compressive strength of concrete is limited. 

### 3.5. Microstructure Analysis of Tunnel Slag Concrete

**Figure 10:**
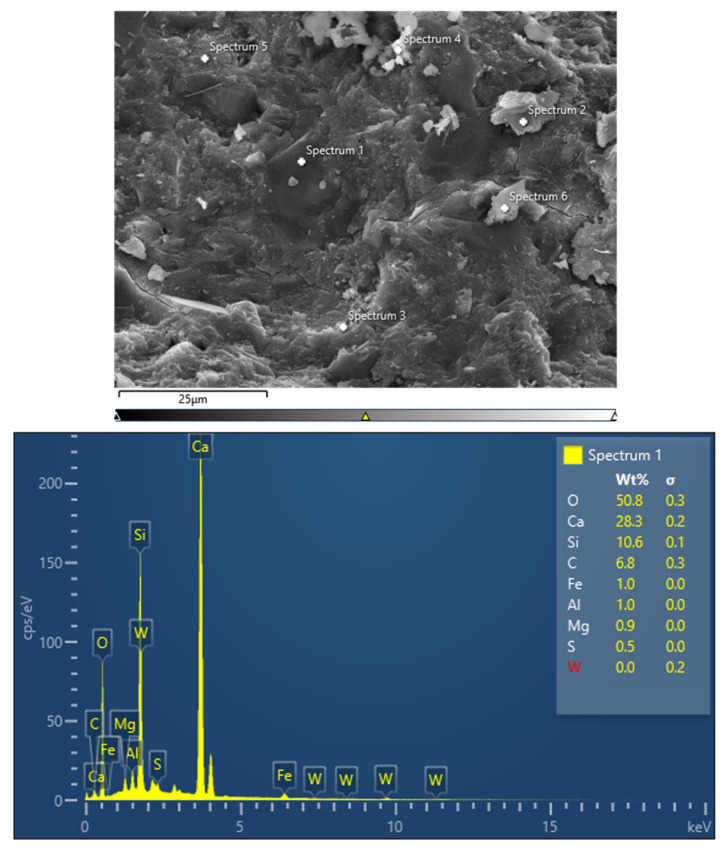
SEM-EDS diagram of tunnel slag concrete specimens.

**Figure 11:**
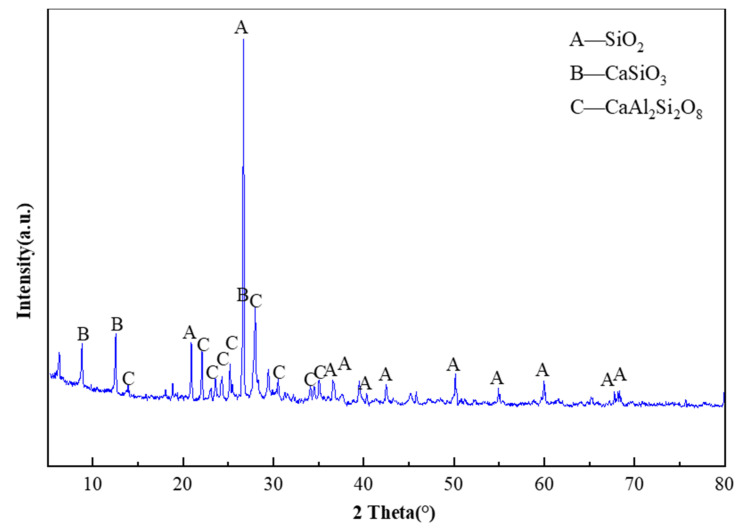
XRD microstructure analysis results.

The microstructure of the material determines the macroscopic properties. Scanning electron microscopy (SEM), energy dispersive spectroscopy (EDS) and X-ray diffractometer (XRD) [38,39,40] physical phase analysis were used to analyze the microstructure of tunnel slag concrete. In turn, the relationship between the microstructure and macroscopic properties of concrete was investigated. The concrete SEM observation and EDS analysis are shown in Figure 10. The results of EDS of tunnel slag concrete are shown in Table 12. XRD microstructure analysis is shown in Figure 11.

From the concrete energy spectrogram and quantitative structural analysis, it can be seen that the elemental compositions of the tunnel slag concrete are mainly O, Si, Ca, Al, C, and Fe. The tunnel slag concrete specimens were tested by XRD. The diffraction pattern was analyzed and the molecular formula and percentage content of the chemical composition of the material constituents were obtained. It was found that the main composition of the tunnel slag concrete samples was SiO_2_, with small amounts of feldspar, calcium silicate, calcium feldspar and calcium cross-zeolite-potassium [41]. Calcium cross zeolite, a kind of zeolite in the main composition of tunnel slag mechanism sand, is chemically composed of SiO_2_ and Al_2_O_3_.On the one hand, under alkaline excitation, zeolite can react with Ca(OH)_2_ precipitated by cement hydration to produce hydrated calcium silicate colloid, which promotes the hydration reaction of cement. On the other hand, zeolite has a lattice-like structure, and its interior is filled with uniformly sized pores and channels. Artificial sand has a large specific surface area after being finely ground by machine, and it can absorb a large amount of water and gas in its natural state and balance with the relative humidity of the atmosphere. When it is mixed into concrete, it can absorb the excess mixing water in concrete and overcome the water retention of concrete. It can make the concrete viscosity increase and increase the amount of aggregate coating. Therefore, it can improve the work performance of concrete.

## 4. Conclusions

With a shortage of natural sand resources, the use of tunnel waste rock as a raw material for sand has become an option for sustainable development. In this paper, a study of the performance of tunnel slag machine-made sand mortar and concrete was carried out through experimental research and gray-correlation analysis. From the experimental results, the following conclusions can be drawn.

1. Machine-made sand mortar flexural and compressive strengths are greater than the same stone powder content of standard sand. The maximum differences of 28-day flexural and compressive strengths are more than 30%. The best stone powder content of the machine-made mortar is in the range of 5% to 8%.
2. By analyzing the performance of the machine-made sand concrete with equal amounts of cement replaced by stone powder, we conclude that the mechanical and working properties of the concrete reach the optimal state when the amount of cement replaced by stone powder is about 6%.
3. The slump of river sand concrete is slightly greater than that of machine-made sand. The compressive strength of machine-made sand concrete is greater with the right amount of stone powder. Under the premise of controlling the amount of stone powder, the tunnel slag machine-made sand concrete has higher strength than river sand concrete.
4. Gray-correlation analysis was used to compare the influencing factors of mechanical properties of concrete. The correlation between the concrete properties and the fineness modulus is the greatest. This points to the direction for the quality control of tunnel slag sand concrete.

This study mainly considered the properties of machine-made sand concrete. Future studies may analyze the effect of stone powder content on the durability performance of machine-made sand concrete.

## Figures and Tables

**Table 1:** Compressive strength test results of parent rock.

Group Number	1	2	3	4	Average Value
Compressive Strength	106.0 MPa	119.1 MPa	129.1 MPa	120.3 MPa	118.6 MPa

**Table 2:** Test of mortar strength of standard sand.

Test Number	Stone Powder Content (%)	Stone Powder (kg/m^3^)	Cement (kg/m^3^)	Standard Sand (kg/m^3^)	Water (kg/m^3^)
S0	0	0.0	450.0	1350	225
S1	1	4.5	445.5	1350	225
S2	2	9.0	441.0	1350	225
S3	3	13.5	436.5	1350	225
S4	4	18.0	432.0	1350	225
S5	5	22.5	427.5	1350	225
S6	6	27.0	423.0	1350	225
S7	7	31.5	418.5	1350	225
S8	8	36.0	414.0	1350	225
S9	9	40.5	409.5	1350	225
S10	10	45.0	405.0	1350	225

**Table 3:** Test of mortar strength of machine-made sand.

Test Number	Stone Powder Content (%)	Stone Powder (kg/m^3^)	Cement (kg/m^3^)	Machine-Made Sand (kg/m^3^)	Water (kg/m^3^)
**M0**	0	0.0	450.0	1350.0	225
**M1**	1	13.5	450.0	1336.5	225
**M2**	2	27.0	450.0	1323.0	225
**M3**	3	40.5	450.0	1309.5	225
**M4**	4	54.0	450.0	1296.0	225
**M5**	5	67.5	450.0	1282.5	225
**M6**	6	81.0	450.0	1269.0	225
**M7**	7	94.5	450.0	1255.5	225
**M8**	8	108.0	450.0	1242.0	225
**M9**	9	121.5	450.0	1228.5	225
**M10**	10	135.0	450.0	1215.0	225

**Table 4:** C35 concrete mix ratio design.

Description	Cement	Fly Ash	Sand	Gravel	Water	Additives
Matching Ratio Design	1.00	0.43	2.56	3.38	0.54	0.017
Amount of Material Per Side/(kg/m^3^)	304	130	777	1029	165	5.21

**Table 5:** Test results of mortar flexural strength of standard sand.

Test Number	3-Day			7-Day			28-Day		
	Average Value (MPa)	Standard Deviation (MPa)	Coefficient of Variation	Average Value (MPa)	Standard Deviation (MPa)	Coefficient of Variation	Average Value (MPa)	Standard Deviation (MPa)	Coefficient of Variation
**S0**	4.7	0.06	1.24%	5.5	0.12	2.11%	7.6	0.26	3.48%
**S1**	4.8	0.06	1.20%	5.5	0.15	2.79%	7.2	0.66	9.11%
**S2**	4.5	0.10	2.22%	5.2	0.15	2.92%	7.4	0.21	2.83%
**S3**	5.1	0.10	1.96%	5.1	0.15	3.02%	7.3	0.25	3.43%
**S4**	4.6	0.06	1.25%	5.3	0.10	1.89%	7.0	0.10	1.43%
**S5**	4.6	0.15	3.30%	5.2	0.15	2.96%	7.3	0.32	4.42%
**S6**	4.5	0.12	2.55%	5.2	0.13	2.57%	7.6	0.21	2.73%
**S7**	4.7	0.10	2.13%	5.2	0.21	4.03%	7.3	0.60	8.30%
**S8**	4.3	0.06	1.33%	4.8	0.10	2.08%	7.2	0.30	4.17%
**S9**	4.4	0.12	2.61%	5.0	0.06	1.16%	7.1	0.25	3.53%
**S10**	4.4	0.15	3.45%	4.7	0.10	2.13%	7.2	0.15	2.11%

**Table 6:** Test results of mortar flexural strength of machine-made sand.

Test Number	3-Day			7-Day			28-Day		
	Average Value (MPa)	Standard Deviation (MPa)	Coefficient of Variation	Average Value (MPa)	Standard Deviation (MPa)	Coefficient of Variation	Average Value (MPa)	Standard Deviation (MPa)	Coefficient of Variation
**M0**	5.7	0.36	6.33%	7.2	0.31	4.26%	9.1	0.31	3.37%
**M1**	6.0	0.44	7.27%	7.4	0.25	3.39%	9.4	0.49	5.23%
**M2**	5.6	0.15	2.74%	6.7	0.15	2.27%	9.0	0.29	3.22%
**M3**	5.0	0.31	6.07%	6.9	0.30	4.35%	9.2	0.45	4.92%
**M4**	5.3	0.21	3.90%	6.5	0.26	4.07%	8.8	0.20	2.27%
**M5**	6.0	0.31	5.06%	6.5	0.31	4.68%	9.7	0.46	4.78%
**M6**	5.8	0.40	7.01%	7.1	0.32	4.55%	9.3	0.26	2.85%
**M7**	6.0	0.47	7.92%	6.5	0.15	2.36%	9.2	0.75	8.21%
**M8**	6.1	0.40	6.59%	7.1	0.26	3.73%	9.8	0.70	7.14%
**M9**	5.4	0.26	4.90%	5.4	0.42	7.76%	8.9	0.35	3.89%
**M10**	5.7	0.17	3.04%	5.9	0.44	7.39%	9.2	0.38	4.13%

**Table 7:** Test results of mortar compressive strength of standard sand.

Test Number	3-Day			7-Day			28-Day		
	Average Value (MPa)	Standard Deviation (MPa)	Coefficient of Variation	Average Value (MPa)	Standard Deviation (MPa)	Coefficient of Variation	Average Value (MPa)	Standard Deviation (MPa)	Coefficient of Variation
**S0**	28.8	0.57	1.98%	37.6	0.47	1.26%	51.8	1.19	2.30%
**S1**	28.5	0.17	0.61%	37.4	0.06	0.15%	49.2	2.00	4.06%
**S2**	27.1	0.06	0.21%	36.5	0.15	0.42%	46.1	1.35	2.93%
**S3**	27.2	0.40	1.48%	35.7	0.15	0.43%	48.3	1.56	3.23%
**S4**	26.1	0.87	3.34%	35.3	0.10	0.28%	49.7	0.85	1.71%
**S5**	25.5	0.15	0.60%	33.6	0.21	0.62%	48.3	2.65	5.49%
**S6**	24.5	0.06	0.24%	32.0	0.06	0.18%	45.9	0.65	1.42%
**S7**	24.9	0.62	2.51%	31.6	0.15	0.48%	43.2	0.96	2.23%
**S8**	24.4	0.12	0.47%	29.9	0.36	1.21%	44.0	1.01	2.31%
**S9**	23.6	0.10	0.42%	29.4	0.10	0.34%	41.4	1.37	3.30%
**S10**	23.5	0.12	0.49%	28.0	0.10	0.36%	43.6	2.31	5.30%

**Table 8:** Test results of mortar compressive strength of machine-made sand.

Test Number	3-Day			7-Day			28-Day		
	Average Value (MPa)	Standard Deviation (MPa)	Coefficient of Variation	Average Value (MPa)	Standard Deviation (MPa)	Coefficient of Variation	Average Value (MPa)	Standard Deviation (MPa)	Coefficient of Variation
**CM0**	29.4	0.35	1.20%	38.2	1.68	4.40%	51.8	0.46	0.89%
**CM1**	29.2	0.76	2.61%	38.7	1.22	3.16%	52.1	0.40	0.77%
**CM2**	30.7	0.56	1.81%	40.0	1.30	3.25%	55.3	0.81	1.46%
**CM3**	30.5	1.30	4.27%	40.7	0.40	0.99%	53.9	0.51	0.95%
**CM4**	30.9	0.78	2.53%	42.2	0.49	1.17%	56.3	1.25	2.22%
**CM5**	31.3	0.75	2.41%	41.8	2.57	6.16%	54.3	1.72	3.17%
**CM6**	30.1	0.93	3.09%	40.6	1.45	3.56%	53.1	1.12	2.10%
**CM7**	29.3	0.20	0.68%	39.4	0.17	0.44%	57.5	0.55	0.96%
**CM8**	28.3	0.10	0.35%	38.7	0.21	0.54%	54.4	1.41	2.59%
**CM9**	27.6	0.15	0.55%	36.3	0.15	0.42%	55.5	1.44	2.60%
**CM10**	25.3	0.15	0.61%	38.2	0.15	0.45%	54.1	0.67	1.23%

**Table 9:** Results of concrete performance tests of stone powder replacing the equivalent amount of cement.

Mixing Amount of Stone Powder (%)	Stone Powder (kg/m^3^)	Cement (kg/m^3^)	Slump (mm)	Slump Expansion (mm)	Compressive Strength (MPa)			
					3-Day	7-Day	28-Day	56-Day
0	0	434	190	490	48.8	55.4	59.4	61.2
3	13	421	190	495	46.4	49.8	59.3	62.6
5	22	412	190	490	47.3	52.5	58.4	60.1
6	26	408	190	485	46.6	51.8	55.5	58.5
9	39	395	185	475	46.1	48.3	53.6	58.8
10	43	391	170	450	45.5	47.1	51.2	53.0
15	65	369	150	430	32.9	40.5	47.0	51.6
20	87	347	130	400	28.2	35.7	40.0	48.0

**Table 10:** Performance test results of river sand concrete with different fineness modulus.

Test Number	Fineness Modulus	Slump (mm)	Slump Expansion (mm)	Compressive Strength (MPa)			
				3-Day	7-Day	28-Day	56-Day
RC1	2.4	220	570	22.5	33.5	38.5	43.2
RC2	2.5	220	540	22.1	32.7	40.8	48.2
RC3	2.6	220	550	22.8	33.1	41.0	47.3
RC4	2.7	230	570	23.2	33.6	42.3	47.3
RC5	2.8	225	555	23.5	33.8	42.1	46.4
RC6	2.9	225	560	23.0	34.4	41.4	46.4
RC7	3.0	205	530	23.3	34.0	41.5	46.5

**Table 11:** Performance test results of machine-made sand concrete with different fineness modulus and stone powder content.

Test Number	Stone Powder Content (%)	Fineness Modulus	Slump (mm)	Slump Expansion (mm)	Compressive Strength (MPa)			
					3-Day	7-Day	28-Day	56-Day
MC1	0	2.4	215	530	29.8	35.9	41.8	45.8
MC2	2	2.4	210	515	29.4	33.3	41.5	45.1
MC3	4	2.4	205	500	29.8	33.2	39.8	49.1
MC4	6	2.4	195	480	28.4	31.9	37.5	42.1
MC5	8	2.4	190	455	25	29.1	36.1	44.7
MC6	10	2.4	175	450	22.2	24.3	35.4	46.6
MC7	0	2.5	215	525	28.9	32.5	42.8	47.3
MC8	2	2.5	210	505	29.2	33.2	41.3	46.8
MC9	4	2.5	205	490	28.3	31.9	42.1	45.6
MC10	6	2.5	200	485	29.5	33.4	40.5	45.2
MC11	8	2.5	185	460	27.5	31.7	38.8	43.2
MC12	10	2.5	180	450	24.2	27.0	37.3	44.1
MC13	0	2.6	220	550	29.5	33.4	43.6	48.5
MC14	2	2.6	215	515	29.3	33.6	43.4	48.1
MC15	4	2.6	210	500	28.7	34.2	42.8	47.4
MC16	6	2.6	200	490	28.9	34.5	40.5	47.1
MC17	8	2.6	195	475	26.1	30.7	37.5	45.1
MC18	10	2.6	180	450	23.4	27.5	36.1	45.7
MC19	0	2.7	220	550	33.5	37.6	44.8	48.4
MC20	2	2.7	220	545	33.0	36.3	44.2	49.7
MC21	4	2.7	220	535	31.4	35.9	43.7	47.8
MC22	6	2.7	210	515	30.7	34.3	42.7	48.7
MC23	8	2.7	205	480	28.5	32.7	38.8	44.7
MC24	10	2.7	180	440	26.1	30.5	37.6	44.8
MC25	0	2.8	225	555	31.8	35.7	45.1	50.6
MC26	2	2.8	215	535	30.5	34.1	45.9	48.7
MC27	4	2.8	215	520	33.3	36.8	44.8	49.1
MC28	6	2.8	205	490	31.5	34.7	42.6	48.2
MC29	8	2.8	195	470	28.4	31.4	39.5	46.2
MC30	10	2.8	190	455	26.8	28.9	37.5	44.3
MC31	0	2.9	225	560	32.2	36.4	44.3	49.6
MC32	2	2.9	215	535	31.1	34.0	46.6	50.0
MC33	4	2.9	205	510	31.5	34.6	44.7	48.5
MC34	6	2.9	200	490	29.9	32.5	42.5	46.9
MC35	8	2.9	190	470	27.3	30.9	41.1	46.8
MC36	10	2.9	185	460	26.4	28.8	36.8	43.6
MC37	0	3.0	205	520	32.8	36.7	45.3	48.8
MC38	2	3.0	205	510	31.5	36.1	45.2	49.4
MC39	4	3.0	205	500	31.3	35.5	44.3	50.7
MC40	6	3.0	200	480	30.8	33.8	43.5	47.3
MC41	8	3.0	195	460	28.6	31.8	41.8	44.1
MC42	10	3.0	180	450	24.3	26.9	34.4	40.4

**Table 12:** Results of EDS analysis of tunnel cavity slag concrete.

Type	O	Na	Mg	Al	Si	S	K	Ca	Fe	Mo	C
Spectrum 1	50.83	-	0.94	1.02	10.61	0.47	-	28.28	1.03	-	6.83
Spectrum 2	45.87	0.15	0.39	0.68	3.79	0.27	-	41.8	0.55	-	6.50
Spectrum 3	66.41	0.23	0.37	2.04	8.00	1.15	0.17	20.4	1.22	-	-
Spectrum 4	60.84	1.05	0.46	2.19	12.38	-	0.46	20.79	0.74	1.09	-
Spectrum 5	56.16	0.46	0.43	2.49	9.85	2.01	0.23	27.26	1.11	-	-
Spectrum 6	58.49	0.25	0.74	2.49	6.57	2.59	0.32	27.34	1.22	-	-

## Data Availability

The data that support the findings of this study are available upon request from the authors.
